# Developing Age-Friendly Cities and Communities: Eleven Case Studies from around the World

**DOI:** 10.3390/ijerph18010133

**Published:** 2020-12-27

**Authors:** Samuèle Rémillard-Boilard, Tine Buffel, Chris Phillipson

**Affiliations:** 1 Research Centre on Aging, CIUSSS de l’Estrie—CHUS, Université de Sherbrooke, Sherbrooke, QC J1K 2R1, Canada; 2 School of Social Sciences, Manchester Institute for Collaborative Research on Ageing (MICRA), The University of Manchester, Manchester M13 9PR, UK; tine.buffel@manchester.ac.uk (T.B.); christopher.phillipson@manchester.ac.uk (C.P.)

**Keywords:** age-friendly cities, ageing, policy, case studies, Global Network of Age-Friendly Cities and Communities, World Health Organization

## Abstract

Developing age-friendly cities and communities has become a key part of policies aimed at improving the quality of life of older people in urban areas. The World Health Organization has been especially important in driving the ‘age-friendly’ agenda, notably through its Global Network of Age-Friendly Cities and Communities, connecting 1114 (2020 figure) cities and communities worldwide. Despite the expansion and achievements of the Network over the last decade, little is known about the progress made by cities developing this work around the world. This article addresses this research gap by comparing the experience of eleven cities located in eleven countries. Using a multiple case study approach, the study explores the key goals, achievements, and challenges faced by local age-friendly programs and identifies four priorities the age-friendly movement should consider to further its development: (1) changing the perception of older age; (2) involving key actors in age-friendly efforts; (3) responding to the (diverse) needs of older people; and (4) improving the planning and delivery of age-friendly programs. The article concludes by discussing the research and policy implications of these findings for the age-friendly movement.

## 1. Introduction

Population ageing is taking place across all countries of the world, raising major issues for the direction of health and social policy. By 2030, two-thirds of the world’s population will be residing in cities, with many urban areas in the developed world having 25 per cent or more of their populations aged 60 and over [1]. The period from the mid-2000s saw a substantial growth in interest in age-friendly issues, with a particular focus on problems facing older people in different types of urban areas [2]. This initial period of development recorded a variety of achievements that stimulated new approaches in areas such as housing, social inclusion, and neighborhood design [3]. However, a combination of widening inequalities within urban environments, together with the impact of austerity on local and municipal government budgets, has raised questions about future progress in age-friendly and related activities. Cities produce many advantages for older people in the form of access to medical services, provision of cultural and leisure facilities, and necessities for daily living. At the same time, they may also create feelings of vulnerability and insecurity arising from high levels of population turnover, environmental problems, and reduced availability of low-cost or affordable housing [4].

This article contributes to the debate on age-friendly cities and communities by, first, reviewing the background to the program of ‘age-friendly cities and communities’ developed by the World Health Organization (WHO); second, reporting on a survey of members of the WHO Global Network of Age-Friendly Cities and Communities; and, third, outlining priorities for the future planning of age-friendly programs.

### Developing Age-Friendly Cities and Communities

The ‘age-friendly’ perspective was first developed by the World Health Organization (WHO) [5,6,7] through a project examining the experiences of older people living in urban environments. The result of this work was a guide identifying the key characteristics of an age-friendly community in terms of service provision (e.g., health services, transportation), the built environment (e.g., housing, outdoor spaces, and buildings), and social aspects (e.g., civic and social participation) [5]. This guide has since become one of the most frequently used tools to assess the age-friendliness of cities and communities [8]. To encourage dissemination of its work, the WHO launched in 2010 the Global Network of Age-Friendly Cities and Communities (GNAFCC), which by the end of 2020 had reached a membership of around 1114 cities and communities in 44 countries.

Any city or community can join the GNAFCC as long as they make a formal commitment to increase their level of age-friendliness. As the WHO [7] (p. 5) indicates, ‘membership of the Network is not a designation but a commitment to making progress on the journey to becoming more age-friendly’. In order to be admitted to the Network participating cities must: (1) agree to share and promote the values and principles of the GNAFCC; (2) commit to developing their work following a 4-step process (i.e., engage and understand, plan, act, measure); and (3) actively participate in the GNAFCC [9].

The Network seeks to support members to become more age-friendly through ‘connecting cities and communities worldwide (via its website, www.agefriendlyworld.com) to facilitate the exchange of information, knowledge and experiences’; ‘inspiring change by showing what can be done and how it can be done’; and ‘supporting cities and communities to find appropriate innovative and evidence-based solutions’ [9] (p. 1). Members of the GNAFCC include communities, cities or other sub-national levels of government located in WHO Member States. In addition to the 1114 participating cities and communities in 44 countries, the Network also has 15 affiliates from 11 countries that play a key role in supporting the Network’s mission. These affiliated programs advocate the work of the Network and advance knowledge and action on age-friendly environments [7].

**Figure 1:**
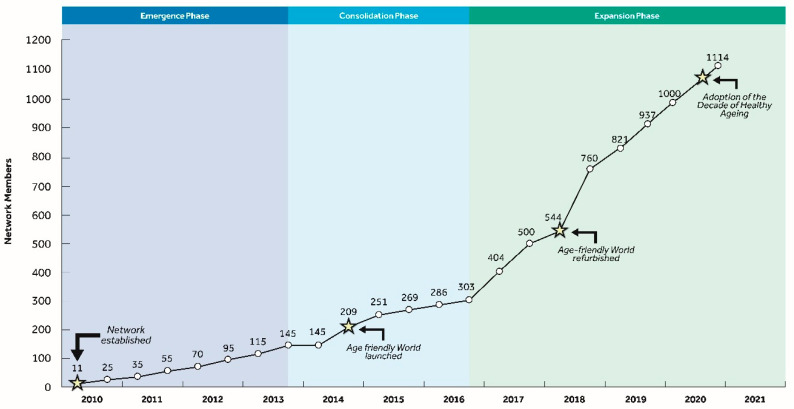
Growth of the Global Network of Age-Friendly Cities and Communities (GNAFCC) (adapted from [7]).

Figure 1 identifies three phases in the growth of the Network: first, the *emergence phase*, characterized by relatively slow initial growth; second, the *consolidation phase* from 2014 to 2016, showing a gradual increase in membership; and third, the *expansion* phase, reflecting a marked acceleration in growth from 2017 to date. Membership of the GNAFCC increased four-fold between 2015 and 2018, with an expansion in the number of affiliates. This is a remarkable finding given continued pressures arising from the impact of economic austerity in many countries around the world. The vulnerability of age-friendly programs, faced with economic austerity, has been further compounded by pressures associated with urban change affecting global as well as de-industrializing cities [10]. Such limitations raise important concerns about the effectiveness and sustainability of age-friendly programs, and whether their growth is likely to continue over the next 10 years.

#### Research on Age-Friendly Issues

The age-friendly movement has begun to attract significant research interest, with groups, for example, in Belgium [11]; Canada [12]; Hong Kong [13]; the UK [14] and the US [15], exploring questions around developing communities responsive to the needs of people as they age. Research in this area has contributed significant knowledge about the ways in which cities are responding to population ageing and building age-friendly communities. Studies have, for example, explored the different steps associated with the age-friendly process, including the planning [16], implementation [17], and evaluation [18] of programs. Researchers have also examined the development of age-friendly initiatives in different contexts, both rural [19] and urban [20], as well as in different countries [21].

Despite these advances in knowledge, few studies have focused on the type of programs developed by partners in the GNAFCC. Ten years after the launch of the WHO network, little is known about the progress of the movement and the experience of cities developing this work around the world. The need to gain more knowledge of age-friendly initiatives has been highlighted by a number of researchers and has been identified by the WHO as a priority for the future of the age-friendly movement [7]. This interest is reflected in the increasing number of tools published to monitor and evaluate the success of programs. For example, the WHO published a guide in 2015 to help cities select the most appropriate indicators for measuring their level of age-friendliness. This document identified a list of ‘core and supplementary indicators’ which cities could choose from and adapt when conducting their own evaluations [22]. Similar tools have also emerged in the age-friendly literature focusing on specific national or local settings (see, for example, [18,23,24]).

The research initiatives identified have yet to provide detailed information on the different types of programs within the GNAFCC. This gap in knowledge reflects a range of methodological challenges linked to the integration of interdisciplinary perspectives, cross-national comparison, measurement, and evaluation [21]. Much of the literature published on the development of age-friendly programs and policies has either been based on a review of the literature or a single case study (of a city or a region). To address some of the limitations of existing work, this paper reports on a multiple case study of eleven members of the WHO Global Network of Age-Friendly Cities and Communities, and addresses the following research questions:

(1) What have been the key goals, achievements and challenges faced by local age-friendly programs since joining the GNAFCC?
(2) What are the key priorities to address to improve the development of age-friendly programs in the future?

## 2. Methodology

The study used a multiple case study approach, one which allows for ‘in depth, multi-faceted explorations of complex issues in their real-life settings’ [25] to compare the experience of eleven members of the GNAFCC. This approach lends itself to capturing information on more exploratory ‘*how*’, ‘*what*’ and ‘*why*’ questions, and has shown to be particularly instrumental in comparing the development of age-friendly programs and policies across different cities [20,26].

**Figure 2:**
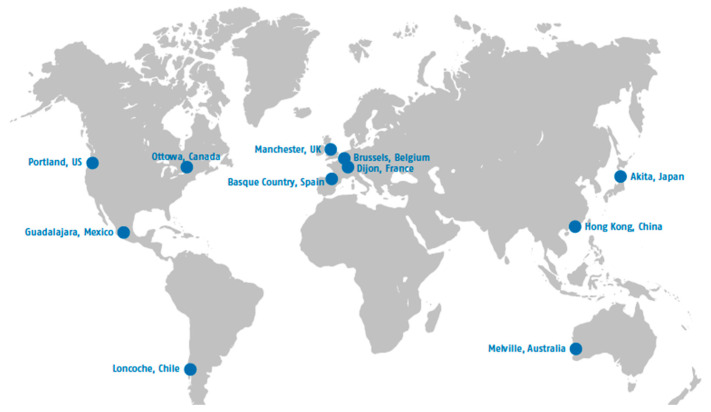
Map of selected cities.

A purposeful sampling strategy was used to identify the eleven cases, with five criteria guiding the selection process [27]. The aim was to select cities that: (1) had an age-friendly program in place at the time of conducting the study; (2) were members of the Global Network of Age-Friendly Cities and Communities; (3) had been involved in the Network either from the beginning or from a relatively early phase of its development; (4) varied in size; (5) were located in different countries. Representatives from the WHO’s Department of Ageing and Life Course assisted in the identification of cases by providing researchers with a list of cities that met the above criteria. The researchers made the final decision and selected the eleven cities presented in Figure 2.

Table 1 summarizes information about the cities which comprised 5 metropolitan areas, 2 medium-size urban areas, 3 small urban areas, and 1 urban cluster [28]. The eleven cities had in most cases joined the GNAFCC in its emergence phase (2010–2013), with the exception of Guadalajara and Loncoche which were admitted to the Network in 2014 and 2016. The percentage of the population aged 65 years and older showed considerable variation, ranging from 9.29% in Manchester to 30% in Akita.

The study was conducted between March 2018 and August 2018. The World Health Organization’s Department of Ageing and Life Course was responsible for inviting selected cities to participate in the study, whilst a research team from the University of Manchester took the lead in collecting data. Three data collection tools were used to document the reality of the eleven cases: questionnaires (*n* = 11), document analysis, and interviews (*n* = 5).

Program representatives who agreed to take part in the study were invited to complete a questionnaire comprising 20 open-ended questions. The questionnaire explored a variety of themes related to the development of age-friendly programs. Participants were asked, for example, to describe the social and demographic contexts in which their program was developed, to identify some of the key objectives and achievements of their program, to identify the key barriers and enablers to developing age-friendly initiatives, and to share their views on the future of their program and the age-friendly movement. They were invited to complete the questionnaire in the language of their choice (English, French or Spanish) and asked to provide as much information as possible on their program and experience, thus allowing substantial amounts of data to be collected on each case.

Eleven questionnaires were completed in total. Each member of the research team was responsible for collecting and analyzing the data related to up to three cities in order to allow them to develop a more in-depth understanding of each case and facilitate communications with participating cities. The researchers contacted participants following the questionnaire completion when any clarifications were required. Five follow-up telephone interviews were also conducted with experts in cities to collect additional information. These interviews were audio-recorded, transcribed verbatim and, where necessary, translated into English.

Finally, a review of the key scientific and grey literature on each program was conducted to enrich the case descriptions. Participants were invited to share any document that would help the researchers develop a better understanding of their program and the context in which it was developed. Additional documents (e.g., baseline assessments, action plans, progress reports) were also drawn from the WHO’s database of age-friendly programs. The content of these documents was not analyzed, per se, but carefully read and used to further our understanding of each case.

The researchers analyzed data relating to the individual cities first, before making comparisons across cases. The questionnaires served as the main source of information for the analysis. Each questionnaire was carefully read and compared with the interviews and the document analysis to clarify certain information, capture the specificities of each program, understand them in their context, and enrich the case descriptions. The results of this phase of analysis were used to write eleven case study reports, each presenting the reality of one age-friendly program. Whilst these reports were written by researchers, the data were mainly drawn from the questionnaires and reflected the views of local representatives. A similar structure was adopted to write the eleven case studies to facilitate cross-case comparisons. Each report provided a detailed description of one case, covering the five following themes: ‘description of the context’, ‘development of the age-friendly program’, ‘impact and evaluation’, ‘opportunities and barriers’, and ‘future of the age-friendly movement’.

A second phase of analysis was conducted to compare the content of the reports and identify common themes across the eleven cases. Each individual report was carefully read and coded line by line using a thematic analysis approach and the qualitative analysis software NVivo. Two questions were used to focus and guide the analysis: (1) What have been the key goals, achievements and challenges faced by local age-friendly programs since joining the GNAFCC? (2) What are the key priorities to address to improve the development of age-friendly programs in the future? This second phase of analysis led the researchers to identify four priorities the age-friendly movement should consider in order to further its development. The next section of this paper reports on the results of the analysis of the 11 age-friendly programs.

## 3. Findings

Four main areas were identified as common across the work of the various age-friendly cities and communities: (1) changing the perception of older age; (2) involving key actors in age-friendly efforts; (3) responding to the (diverse) needs of older people; and (4) improving the planning and delivery of age-friendly programs. Each of these is presented and discussed in more detail in the following sections.

### 3.1. Changing the Perception of Older Age

All participants highlighted the importance of age-friendly programs *challenging the negative image and portrayal of ageing*. The need to shift perception, change mindsets and promote a more positive vision of ageing was identified as a key priority across the eleven cases. Representatives from Akita, for example, considered it important to ‘create a society where people consider the 100-year life as a positive opportunity’; representatives from Dijon to ‘remind people that living longer lives is a chance’; and representatives from Manchester to ‘change the narrative to one that celebrates the valuable role and contribution of older people’. A variety of projects have been developed in participating cities to promote a more positive vision of ageing. One popular way to achieve this goal has been to encourage the development of communication campaigns that use more realistic and non-stereotypical images of ageing, a more positive language, and paint a more diverse portrait of the older population. Cities had also developed communications tools, such as logos, symbols, charters, and slogans to promote age-friendly initiatives and challenge the negative image of ageing. The city of Portland, for example, applied the slogan ‘nothing about them, without them’ in its communications to describe the city’s age-friendly approach and promote the civic engagement of older people.

Promoting the social participation of older people was also seen as a way to challenge ageism, by making this group more visible ‘and [making older people] seen as active and essential members of the community’ (Loncoche). Participating cities worked toward this goal in two main ways: first, by providing older people with more opportunities to take part in social activities; and second, by involving them more directly in age-friendly interventions. A variety of activities, projects and services aimed at increasing the social participation of older people were created in the eleven cities following their admission to the GNAFCC. One of the key achievements of the age-friendly program in Brussels, for example, has been the opening of seven social meeting spaces for older people across the city. Called ‘Spaces S’ (for seniors), these offer leisure activities, information, sports, and training sessions for older people that are delivered directly at the neighborhood-level. Similar places have also been created in Dijon and Loncoche and are considered as key resources for the community. Another popular way to promote the participation of older people has been through the formation of representative bodies of different kinds. Various initiatives (e.g., activities, consultations, studies) and mechanisms (e.g., committees, boards, roundtables) have been established in order to give older people a more central role in age-friendly developments. Whilst older people’s levels of participation may have differed from one city to another, the need for their expertise to shape the development of age-friendly programs was recognized and considered as a key priority in all cities.

The age-friendly program was also used *to raise awareness of key issues and concerns facing older people*. This was viewed as a way of promoting both a vision of older age and improving the treatment of older people. Participating cities had adopted various strategies to achieve this goal. The city of Guadalajara, for example, had developed training courses for the public sector to help employees become more aware and sensitive towards the specific needs of older people. The cities of Portland and Manchester worked closely with university researchers and held conferences to report on current research and practices on ageing which could feedback to work within the local community. Intergenerational initiatives had also been developed as another approach to raising awareness. Encouraging younger and older people to interact on a more regular basis was seen as way to ‘promote the exchange of knowledge’ (Guadalajara); ‘tackle negative stereotyping of older people’ (Guadalajara); and ‘increase respect for older generations’ (Loncoche).

Responses from the survey suggested that age-friendly initiatives have helped challenge negative perceptions of ageing, with awareness of the existence of these often a driving force behind the development of programs. When asked to describe the impact of their program, a majority of participants reported that this work had contributed to making older people more visible in their city and raised awareness of their views and needs amongst both the population and service providers. The representative from Loncoche, for example, mentioned that the ‘perception of older age was changing’ in their city; Guadalajara commented that local actors were now ‘more aware of the issues surrounding ageing’; and the representative from Ottawa that their program had ‘increased awareness of older people’s needs and realities’. Despite this progress, changing the perception of older age remained a key concern of respondents. Combating ageism was described not only as an important priority to address to improve the quality of life of older people, but also the delivery of age-friendly programs.

Ageism—and more specifically the lack of interest relating ageing issues—was identified as one of the key *barriers* to developing age-friendly work. Representatives from Dijon went so far as to describe ageism as a ‘*blockage*’ and as ‘*the principal obstacle the city had to face’*. The difficulty to get traction for ageing issues can be an important barrier to the development of age-friendly initiatives given the wide variety of demands on municipal budgets—not least given new pressures such as those associated with COVID-19. Raising awareness of the needs of older people amongst service providers will be essential to secure more support for developing age-friendly activities.

### 3.2. Involving Key Actors in Age-Friendly Efforts

The second major theme to emerge from the research was the need to involve key actors in age-friendly projects. When asked to comment on the progress of their work, participants often referred to the development of new partnerships and collaborations as a key goal for the future of their programs. Establishing links with a wide range of actors (e.g., local councils, community organizations, businesses, universities, older people), working in a variety of domains (e.g., housing, transport, health, urban planning, social work, environment), was identified as a key success factor for the development of age-friendly initiatives. The research showed that the cities were able to secure the support of a variety of stakeholders and develop innovative partnerships as part of their work. Akita and Melville, for example, were especially successful in gaining the involvement of businesses. At the time of conducting the research, 88 organizations from the private sector were registered as ‘Age-Friendly Partners’ and involved in making the city of Akita more age-friendly. Guadalajara, Manchester, Ottawa, and Portland had worked closely with universities and researchers throughout the development of their programs. Portland State University played a leading role in developing age-friendly work in the city, assuming both co-ordination and leadership roles.

Cities had developed a variety of mechanisms to facilitate these collaborations. Dijon, for example, created an innovative platform called ‘*l’Observatoire de l’Âge’*. This participatory mechanism brings together 83 members (2018 figures) from various groups (i.e., 10 elected officials; 39 local residents; 9 neighborhood representatives; 6 retiree representatives; 4 institutional partners; 10 professional experts; and 5 researchers), divided into work committees. Each committee is allocated a specific theme and asked to develop concrete propositions and projects to improve an ageing issue during the year. As Dijon’s representatives explained, this way of working was considered beneficial for the program because it ‘encourages stakeholders to compromise and prioritize’. At the time of conducting the study, the cities of Brussels, Manchester and Ottawa were respectively working in close collaboration with a ‘Senior Advisory Council’, an ‘Older People’s board’ and a ‘Senior Roundtable’ all comprised of older residents, to shape the development of their program.

The research also highlighted the need to involve actors working at different levels in age-friendly programs. As the movement progresses, scaling up projects and establishing collaborations with actors working at the local, regional, and national level emerged as a growing concern for participating cities. This appeared especially important for large metropolitan areas such as the Basque Country, Hong Kong, and Manchester, which were developing their work at the regional level. More than 50 municipalities had joined the age-friendly movement in the Basque Country, with support from the Department of Employment and Social Policies, and the Matia Gerontological Institute. Melville identified work with the Government of Western Australia as one of the key achievements of its program, whilst the cities of Brussels and Dijon expressed an interest in collaborating with organizations working at the regional and national level to conduct projects on specific themes (e.g., social exclusion and social isolation).

A large majority of participants referred to the development of new collaborations—and the strengthening of existing partnerships—as two of the key achievements of their program. Respondents considered that these collaborations brought important benefits to their work, including the possibility for their program to ‘benefit from the expertise of a variety of actors’ (Portland); to ‘develop a wider range of initiatives’ (Manchester); to ‘involve the voices of different groups‘ (Melville); to ‘look at ageing issues from different angles’ (Dijon); to make a variety of actors ‘see the importance of becoming age-friendly’ (Akita); and to ‘improve the dialogue between the city council and citizens’ (Basque Country). Despite this progress, involving key actors in age-friendly efforts was seen as a challenge and considered as an important priority to address for the future of the age-friendly movement. The research found that certain actors remained difficult to involve in age-friendly efforts, especially given budgetary pressures, and competing economic and social priorities [2]. Participants believed they could achieve more with their program if more actors considered their work through ‘an ageing lens’ and expressed their wish ‘for the age-friendly approach to become an automatic consideration in all plans and work’ (Manchester) of their city in the future. The lack of interest of certain actors for ageing issues was, however, seen as an obstacle to achieving this goal, reinforcing the idea that raising awareness and challenging the negative perception of older age amongst service providers would be essential for the age-friendly movement to achieve its full potential.

### 3.3. Responding to the (Diverse) Needs of Older People

The third priority to emerge from the research was the need to respond to the (diverse) needs of older people. The research highlighted the need for age-friendly programs to address a variety of themes and domains in order to achieve this goal. Projects aiming to improve the social environments had been especially popular across the eleven cities. Cities had worked on a wide range of issues since launching their programs, ranging from attempts to increase the level of employment in the older working population, to projects aimed at widening participation in arts and cultural activities. Manchester launched a program called ‘the Age-Friendly Manchester Culture Program’ which brought together 19 cultural organizations (e.g., museums, orchestras, theatres) from across the city with the aim of making arts and culture more accessible to older people. In 2018, more than 150 older volunteers were involved in one of its flagship projects (the ‘Culture Champions’ scheme), acting as cultural ambassadors in their community.

Another priority for action amongst the cities has been in the area of health promotion. A variety of initiatives have been developed to promote healthy and active ageing, as well as sports and physical activity in older age. Guadalajara developed a program called ‘Taking Control of your Health’ which sought to encourage healthier eating, physical activity, and healthy cognitive function amongst the older population. The project set a target of reaching 2400 people by the end of 2018. Improving the built environment also emerged as an important area of concerns, with transport and mobility being amongst the themes most frequently addressed. Since joining the GNAFCC, cities have, for example, installed more seating, benches, and lighting in public spaces, and improved pavements and roads to make them safer for older people. They have also developed projects to increase the accessibility of transport systems, public buildings, and specific areas in their city. Dijon, for example, has worked on increasing the age-friendliness of its city center for older people, going as far as to pedestrianize certain areas to increase their accessibility.

Respondents to the survey reported that these projects had had a positive impact on their older population. When asked to describe the key benefits of their programs, participants explained that this work: had allowed them to ‘reach a large number of older people’ (Guadalajara); had contributed to ‘making the community a better place in which to age physically, socially, and economically’ (Portland); had ‘led to concrete changes in municipal operations, policies and communications’ (Ottawa); and had ‘increased the accessibility and affordability of programs and services’ for older people in their city (Ottawa). Whilst age-friendly programs had undoubtedly led to the development of important and innovative initiatives, participants expressed the wish to raise the ambition of their program in the coming years, albeit aware for constraints on budgets given a climate of economic austerity.

Meeting the needs of diverse groups within the older population was also highlighted by a number of respondents. Representatives from Brussels, for example, highlighted the importance (and difficulty) for the age-friendly program to respond to the needs of different age groups within the older population: ‘the 55–65 years old, the 65–80 years old and the 80 and over’. Representatives from Hong Kong and Akita—recognized for the rapid ageing of their populations—expressed a similar concern and stressed the importance of developing initiatives to support the ‘oldest-old’. Dijon highlighted the need for age-friendly initiatives to support vulnerable groups, and ‘the groups the most at risk of being isolated, such as people with disabilities’; Melville highlighted the need to develop more initiatives for people living with dementia; and the cities of Manchester and Ottawa—considered as ethnically diverse cities—the importance of projects that recognized older people’s diverse cultural backgrounds. Manchester, for example, had developed a substantial program of work tackling issues relating to social isolation, linking with a range of organizations representing older people from Black, Asian, and Minority Ethnic (BAME) groups.

These findings illustrate both the importance and complexity of meeting the needs of a rapidly ageing population. In order to increase the age-friendliness of their cities, age-friendly programs must not only address a variety of themes but also address the needs of different groups of older people. Achieving this goal is complex, and even more challenging that age-friendly programs are developed with limited resources. The lack of resources was identified as one of the key barriers to developing age-friendly initiatives. The research found that age-friendly programs were often developed in a context of financial restraint and cuts to public services, with insufficient budget and limited staff, forcing age-friendly programs to prioritize between a wide range of issues and limiting the scope of their work. Securing more resources for age-friendly initiatives will be essential to raise the ambition of age-friendly programs and meet the needs of a wider group of older people. As representatives from Loncoche explained, whilst age-friendly programs ‘can be successful with limited resources [...] some projects require more substantial investments in order to achieve their aims’.

### 3.4. Improving the Planning and Delivery of Age-Friendly Programs

A fourth major theme to emerge from the research was the need to improve the planning and delivery of age-friendly programs. When asked to comment on the progress of their program, a majority of participants referred to the organizational dimension of their work and reflected on ways to improve its development. Cities highlighted the need for age-friendly programs to inform their planning by identifying the key priorities to address in their city. Conducting a baseline assessment and collecting data to better understand ‘what communities need and want’ (Melville) was often considered as the first step of the planning process. A variety of initiatives were developed across the cities to achieve this goal. The city of Hong Kong, for example, conducted focus groups with 96 participants drawn from a wide range of stakeholders to orient the development of its program, whilst the city of Brussels chose to take part in a research project called ‘the Belgian Ageing Studies’ to collect data on the needs of their older population and inform its decision-making [29]. Cities have also shown an interest in documenting the needs of a wide range of groups within their older population. The city of Ottawa, for example, conducted 24 public consultation sessions with older people to inform the development of its first age-friendly action plan. Of this number, nine targeted the general population but took place in different locations (allowing urban, rural, francophone and anglophone older residents to be represented) and 15 were designed for older adults with unique needs (e.g., aboriginal elders, natural caregivers, isolated older adults, LGBTQ+ community). Participants highlighted the importance for age-friendly programs to be informed by knowledge and their wish to collect more data to inform their decision-making in the future. The lack of resources was, however, often seen as an obstacle to achieving this goal.

Selecting the type of project to implement also emerged as a key step of the planning process. The research suggested that participating cities had addressed age-friendly issues in different ways since joining the GNAFCC. First, cities had developed new projects and services to increase their level of age-friendliness. These varied in scope and addressed different themes, ranging from promoting the social participation of older people to making public spaces safer and more accessible. Second, cities had developed new ways of working, established innovative partnerships, and created new platforms and mechanisms to facilitate these collaborations. Third, cities had worked on influencing policy and making long-term changes in certain domains. Portland’s approach, for example, had been especially focused on shaping policy for the future. As program representatives explained, this choice was made ‘intentionally to create sustainable, long-term change that will benefit both current and future generations’. Their efforts allowed for a set of age-friendly policies to be incorporated into Portland’s 2035 Comprehensive Plan, a key document which took effect in 2018.

The research indicated that sharing best practices could help cities identify the most beneficial projects to develop in their city. Local representatives saw positively the possibility to take part in knowledge exchange activities. Participants highlighted the need for cities and organizations ‘to share best practices’ (Ottawa); ‘to learn both from failures and successes’ (Akita); ‘to identify barriers to implementation and develop solutions’ (Guadalajara); and ‘to showcase their innovations and experimentations’ (Dijon) as the age-friendly movement develops. The GNAFCC was seen as a key platform for achieving this goal. When asked to reflect on the future of the age-friendly movement, participants highlighted the important role the Network played in connecting participating cities. The possibility to interact with other cities was considered particularly helpful for communities less advanced in their efforts to become more age-friendly and seen as a way to encourage more cities to join the age-friendly movement.

Securing more political support was also seen as a way to improve the planning and delivery of age-friendly programs. The possibility to rely on strong political support was identified as a key success factor for the development of age-friendly initiatives. Manchester representatives, for example, identified the ‘political support and advocacy from the City’s leadership’ and ‘the commitment from services and organizations’ as two of the key success factors for developing their program. Ottawa’s representatives shared similar views and explained that the strong support they received from political leaders in their city ‘provided strength, momentum, credibility and enabled staff buy-in’ for their program. As their programs continue to expand, several cities expressed a wish to secure more support for developing this work and involve more city departments in their age-friendly efforts.

Evaluating the success of age-friendly programs finally emerged as a priority for the future of the age-friendly movement. Participants all recognized the importance of evaluating their work. A large majority of them were reflecting on ways to assess the impact of their program at the time of conducting the study. The city of Ottawa, for example, was working on the development of an Age-Friendly Evaluation Framework and had analyzed ‘baseline data for 66 indicators covering medium and longer-term outcomes and impacts across the 8 areas of an age-friendly community’. The city of Manchester was developing ‘a set of statistical indicators to measure the progress and impact of its work’, working in close collaboration with a national foundation on ageing. Despite this progress, the research found that limited evaluation had been carried out in participating cities, but that demonstrating the impact of age-friendly work will be important to raise the ambition of age-friendly programs and ensure their sustainability. As Portland representatives explained, collecting more data could be beneficial for age-friendly initiatives and help ‘provide the impetus for the work and support for continuation’.

## 4. Discussion

This article set out to reflect on the progress of the age-friendly movement by comparing the experience of eleven participating cities in eleven countries. More specifically, it aimed to explore the key goals, achievements, and challenges of age-friendly programs and some of the key cross-cutting themes. The paper identified four priorities to further the development of the age-friendly movement. First, it highlighted the continuing need to change the perception of older age amongst service providers. Despite the progress made by cities, the article showed that promoting ageing remained a challenge for several years after the launch of age-friendly programs. It also showed that tackling ageism was not only important in improving the quality of life of older people, but also in enabling the delivery of age-friendly programs themselves. Gaining prominence for ageing issues can be challenging when there are competing priorities for limited resources. Raising awareness of the needs of older people will be important for age-friendly issues to remain on the political agenda of their city and to ensure the sustainability of local age-friendly programs on the long term.

Second, it highlighted the need to involve key actors in age-friendly projects. The study showed that, on the one hand, mainstreaming ageing was essential to increase the age-friendliness of cities; on the other hand, that certain stakeholders resist direct involvement in age-friendly initiatives. Reflecting on ways to convince actors from different domains and working at different levels to take part in age-friendly efforts will be essential to address the diverse needs of older people. Whilst the importance of developing cross-sectoral collaborations has been acknowledged by a number of researchers in the age-friendly literature, little is known about the key enablers and barriers to establishing these partnerships and the most effective mechanisms to facilitate collaborations. As it continues to expand, the age-friendly movement would benefit from reflecting and conducting more research on these topics.

Third, the survey confirmed the need for age-friendly programs to respond to the (diverse) needs of older people. As this study illustrates, increasing the age-friendliness of cities requires responses to the varied needs of different groups of older people. Whilst they have made significant progress, participating cities all expressed a wish to develop more ambitious projects and reach new groups within the older population in the future, for example those from minority communities, and those living in areas of multiple deprivation. This raises the issue of the extent to which age-friendly programs can tackle wider economic and social inequalities within society, these having become more prominent over the past 10 years [2,10]. In this regard, if success is to be achieved for the kind of initiatives described in this paper—partnerships will be needed with a range of movements seeking to improve the lives of marginalized and excluded groups within cities. This challenge raises important concerns regarding the capacity of cities to achieve this goal with what are often limited resources. Finding ways to secure additional support will be important for the age-friendly movement to achieve its full potential. Linking age-friendly issues to other priorities (or age groups) within cities, for example, might help local actors get more traction for the age-friendly agenda. Establishing partnerships with other social movements might also have benefits, notably around areas such as climate change, and campaigns around rights to the city. Collecting more data and conducting more evaluation might also help demonstrate the benefits of developing this work and help secure the support of influential stakeholders.

Fourth, the survey highlighted the need to improve the planning and delivery of age-friendly programs. Given the large number of needs to address and the limited resources allocated to develop this work, cities are forced to prioritize between a wide range of issues. Identifying the most important challenges to address in their city and deciding how to tackle them requires careful planning. This study suggests that offering programs representatives more opportunities to take part in knowledge exchange activities and share best practices could support their planning and help them select the most optimal projects to develop in their city. As the movement continues to expand, participating cities might also benefit from having access to more tools and information on how to best organize and structure the development of their program. Platforms such as the GNAFCC, or its affiliated programs, can play an important role in this respect by connecting cities and facilitating the sharing of information [7].

Finally, the study suggests that these four priorities overlap and influence each other. Raising awareness of the needs of older people, for example, can help convince more actors to invest in age-friendly efforts, which can, in turn, improve the delivery of age-friendly programs and allow age-friendly initiatives to be more ambitious and better respond to the diverse needs of their population.

### Limitations of the Research

Although the study identifies important priorities to address to further the development of the age-friendly movement, the research also has limitations. First, selected cases may not be representative of the whole age-friendly movement. The choice to select more experienced cities was made to allow for a more in-depth reflection on their progress. Whilst their achievements may not represent those of less advanced cities, the challenges and priorities discussed in this paper are likely to speak to any city or community involved in developing this work. There is to date limited information on age-friendly initiatives within low-income countries, and the specific challenges which they face. A research initiative to review issues relating to the scope, implementation, and barriers faced by age-friendly work in this context, is urgently needed. Second, the analysis drew mainly on questionnaires completed by local representatives, and therefore, strongly reflected their perception of their program. Whilst these participants were considered better able to comment on the development of their program and identify the key success factors and challenges to developing age-friendly initiatives, their views may not represent that of other actors in their city. Interviewing a variety of actors, and including the voice of older people in particular, would have allowed the research to paint a more nuanced portrait of each program and develop a more in-depth understanding of each case. Despite these limitations, this exploratory study makes an important contribution to the age-friendly literature by comparing the experience of eleven local age-friendly programs in eleven countries. To our knowledge, this study is the first to compare the experience of that many cities as part of a single research project. This study highlights the need to conduct more empirical, comparative, and cross-national studies on age-friendly initiatives. It also demonstrates the potential of such studies to provide pointers for future research and policy developments on age-friendly issues.

## 5. Conclusions

This article makes a contribution to the age-friendly literature by identifying four priorities the age-friendly movement should consider to further its development: first, challenging the negative perception of older age and raising awareness of the needs of older people; second, involving key actors in age-friendly programs; third, responding to the (diverse) needs of older people; and fourth, improving the planning and delivery of age-friendly programs. The study also highlighted the benefits of conducting more empirical, comparative, and cross-national studies to better understand the development of the age-friendly movement. As it continues to expand, measuring the progress of the age-friendly movement—and documenting the experience of participating cities—will be essential to demonstrate the benefits of the age-friendly approach, and directions for future policy and practice. Such activity will be especially important in a context of increasing pressure on local authority and municipal budgets given a combination of cuts to the funding of social programs and the impact of the COVID-19 pandemic in amplifying social inequalities [30]. However, it is precisely in this situation that the benefits of adopting an age-friendly approach may become apparent. In particular, in helping to ensure that support for older people is maintained and that the voices of those growing old continue to be heard and acted upon.

## Figures and Tables

**Table 1:** Key characteristics of the eleven cases.

	City (or Region)	Country	Population	GNAFCC Admission	% of People Aged 65 and Over
Metropolitan areas	Hong Kong	China	7.3 million	2010	14.7
	Basque Country	Spain	2.2 million	2013	22
	Guadalajara	Mexico	1.5 million	2014	10.5 *
	Ottawa	Canada	934,243	2011	15.4
	Portland	USA	647,805	2010	11.6
Medium-size urban areas	Manchester	UK	541,300	2010	9.29
	Akita	Japan	310,407	2011	30
Small urban areas	Brussels	Belgium	179,277	2010	11.1
	Dijon	France	155,114	2010	22.1 *
	Melville	Australia	102,252	2010	18.6
Urban cluster	Loncoche	Chile	23,612	2016	14

* % of people aged 60 and over.

## Data Availability

Restrictions apply to the availability of these data. Data was obtained through the Word Health Organization (Department of Ageing and Life-Course) and are available from the authors with the permission of the World Health Organisation (Department of Ageing and Life-Course).

## References

[1] Organisation for Economic Co-operation and Development (2015). Ageing in Cities.

[2] Buffel T., Phillipson C. (2016). Can global cities be ‘age-friendly’ cities? Urban development and ageing populations. Cities.

[3] White S., Hammond M., Buffel T., Handler S., Phillipson C. (2018). From representation to active ageing in a Manchester neighbourhood: Designing the age-friendly city. Age-Friendly Cities and Communities: A Global Perspective.

[4] Hertz N. (2020). The Lonely Century: Coming together in a World that’s Pulling Apart.

[5] World Health Organization (2007). Global Age-Friendly Cities: A Guide.

[6] World Health Organization (2015). World Report on Ageing and Health.

[7] World Health Organization (2018). The Global Network for Age-Friendly Cities and Communities: Looking Back over the Last Decade, Looking Forward to the Next.

[8] Plouffe L., Kalache A., Voelcker I., Moulaert T., Garon S. (2016). A critical review of the WHO age-friendly cities methodology and its implementation. Age-Friendly Cities and Communities in International Comparison. Political Lessons, Scientific Avenues, and Democratic Issues.

[9] World Health Organization Membership in the Global Network of Age-friendly Cities and Communities (GNAFCC). https://www.who.int/ageing/age-friendly-environments/GNAFCC-membership-en.pdf.

[10] Buffel T., Phillipson C. (2018). A manifesto for the age-friendly movement: Developing a new urban agenda. J. Aging Soc. Policy.

[11] Moulaert T., Houioux G., Moulaert T., Garon S. (2016). A Belgian case study: Lack of age-friendly cities and communities knowledge and social participation practices in Wallonia. Age-Friendly Cities and Communities in International Comparison. Political Lessons, Scientific Avenues, and Democratic Issues.

[12] Garon S., Veil A., Paris M., Rémillard-Boilard S., Moulaert T., Garon S. (2016). How can a research program enhance a policy? AFC-Quebec governance and evaluation opportunities. Age-Friendly Cities and Communities in International Comparison. Political Lessons, Scientific Avenues, and Democratic Issues.

[13] Phillips D.R., Woo J., Cheung F., Wong M., Chau P.H., Buffel T., Handler S., Phillipson C. (2018). Exploring the age friendliness of Hong Kong: Opportunities, initiatives and challenges in an ageing Asian city. Age-Friendly Cities and Communities: A Global Perspective.

[14] Buffel T., Handler S., Phillipson C., Buffel T., Handler S., Phillipson C. (2018). Age-friendly cities and communities: A manifesto for change. Age-Friendly Cities and Communities: A Global Perspective.

[15] Greenfield E.A., Oberlink M., Scharlach A.E., Neal M.B., Stafford P.B. (2015). Age-friendly community initiatives: Conceptual issues and key questions. Gerontologist.

[16] Greenfield (2018). E.A. Getting started: An empirically derived logic model for age-friendly community initiatives in the early planning phase. J. Gerontol. Soc. Work.

[17] McDonald B., Scharf T., Walsh K., Buffel T., Handler S., Phillipson C. (2018). Creating an age-friendly county in Ireland: Stakeholders’ perspectives on implementation. Age-Friendly Cities and Communities: A Global Perspective.

[18] Buckner S., Pope D., Mattocks C., Lafortune L., Dherani M., Bruce N. (2017). Developing age-friendly cities: An evidence-based evaluation tool. J. Popul. Ageing.

[19] Menec V., Bell S., Novek S., Minnigaleeva G.A., Morales E., Ouma T., Parodi J.F., Winterton R. (2015). Making rural and remote communities more age-friendly: Experts’ perspectives on issues, challenges, and priorities. J. Aging Soc. Policy.

[20] Buffel T., Rémillard-Boilard S., Walsh K., McDonald B., Smetcoren A.-S., De Donder L. (2020). Age-friendly approaches and old-age exclusion: A cross-city analysis. Int. J. Ageing Later Life.

[21] Moulaert T., Garon S. (2016). Age-Friendly Cities and Communities in International Comparison. Political Lessons, Scientific Avenues, and Democratic Issues.

[22] World Health Organization (2015). Measuring the Age-Friendliness of Cities: A Guide to Using Core Indicators.

[23] Chapon P.-M., Lefebvre P.-O., Philipona A., Finot F. (2015). Measuring the impact of an “age-friendly city” approach on a territory by setting up cross-cutting indicators. Gériatrie Psychol. Neuropsychiatr. Vieil..

[24] Dikken J., van den Hoven R.F.M., van Staalduinen W.H., Hulsebosch-Janssen L.M.T., van Hoof J. (2020). How older people experience the age-friendliness of their city: Development of the Age-Friendly Cities and Communities Questionnaire. Int. J. Environ. Res. Public Health.

[25] Crowe S., Cresswell K., Roberston A., Huby G., Avery A., Sheikh A. (2011). The case study approach. BMC Med. Res. Methodol..

[26] Rémillard-Boilard S. (2019). Developing Age-Friendly Cities: A Public Policy Perspective. Doctoral Thesis.

[27] Patton M.Q. (2015). Qualitative Research & Evaluation Methods: Integrating Theory and Practice.

[28] Organisation for Economic Co-operation and Development (2012). Redefining “Urban”: A New Way to Measure Metropolitan Areas.

[29] De Donder L., De Witte N., Verté D., Drury S., Buffel T., Smetcoren A.S., Brosens D., Verté E. (2014). Developing Evidence-Based Age-Friendly Policies: A Participatory Research Project.

[30] Buffel T., Doran P., Goff M., Lang L., Lewis C., Phillipson C., Yarker S. (2020). Covid-19 and inequality: Developing an age-friendly strategy for low-income communities. Qual. Ageing Older Adults.

